# How Does COVID-19 Pandemic Influence the Sense of Belonging and Decision-Making Process of Nursing Students: The Study of Nursing Students’ Experiences

**DOI:** 10.3390/ijerph17155603

**Published:** 2020-08-03

**Authors:** Luis Miguel Dos Santos

**Affiliations:** Woosong Language Institute, Woosong University, Daejeon 34514, Korea; luismigueldossantos@yahoo.com or luisdossantos@woosong.org

**Keywords:** COVID-19 pandemic, nursing human resources, nursing shortages, nursing workforce, Social Cognitive Career Theory

## Abstract

Financial consideration, internal and external influence, personal goal, and educational achievement always influence the decision-making process and behavior of individuals. Using nursing students as the population, the researcher employed the Social Cognitive Career Theory as the theoretical framework to examine the nursing human resources shortages and how would the COVID-19 pandemic influence the experiences, sense of belonging, and career decision-making process of 58 nursing students in South Korea. The researcher categorized the sharing into two groups, which were before the influence of the COVID-19 pandemic, and the changes during the COVID-19 pandemic. The results indicated that financial consideration was the significant reason why South Korean nursing students decided to study nursing regardless of the COVID-19 pandemic. More importantly, almost all participants decided to leave the nursing profession due to the COVID-19 pandemic and the consideration between financial factor and personal sacrifice. The outcomes of this study provided a blueprint for human resources professionals, government leaders, policymakers, school leaders, and hospital managers to reform their current curriculum and human resources planning to overcome the potential human resources gaps in the soon future due to the COVID-19 pandemic.

## 1. Introduction

The medical and nursing professions have faced significant human resources shortages for more than a century [1,2]. Many regions and nations have established and invested in a large number of pre-service nursing training and education programs. Every year, as a lifelong career investment, a large number of secondary school graduates decide to enter a pre-service nursing training and education program in order to become registered nurses. However, social, cultural, financial, personal, environmental, internal, and educational perspectives and concepts may change the experiences, sense of belonging, and career decision-making process of individuals. For many people, and particularly for secondary school graduates without working experience, deciding on a career pathway and a university major is one of the hardest steps [3]. Therefore, a number of university students may select the wrong university major and career pathway for them as their experiences and selections during secondary school may not match their current expectations and lifelong career investment [4].

### 1.1. Literature Review

Although nursing is one of the most popular university majors among both traditional-age and returning students, for various reasons (e.g., environmental factors, social influences), many students may find that nursing training and education programs do not match their personal goals and expectations [5]. A previous study [6] indicated that nursing students usually select their major due to social and cultural expectations. Unlike westernized society, in the East Asia region, university major selection and even career pathway are the results of parental recommendations and the outcomes of social expectations. The results of this study indicated that 48 nursing students were forced to study for a nursing degree due to the expectations of their parents and school counselors. Due to the mismatch between their university major and their personal goals and expectations, all of these nursing students stated that they would not be entering the medical or nursing profession after they graduated from university. Such mismatching of human resources always produces harmful results as both students and government agencies invest resources in training [7].

Another study [8] found that the nursing profession is not a short-term career development but rather a lifelong investment. Unlike other business environments, where individuals may change companies and working environment on the basis of contemporary business developments and personal interests, registered nurses usually work in similar working environments, such as hospitals, medical centers, and clinics. Although second-career developments are allowed, most nurses do not tend to change their career pathways due to the meaningful nature of their position [9].

Job nature is another consideration in individuals’ career development in the field of nursing. A recent study [10] indicated that student drop out is one of the biggest problems for nursing school leaders. Although nursing students need to pass an admission interview in order to enter a nursing training and education program, many still do not understand the nature, responsibilities, workload, and even administrative work of the job. Therefore, when nursing students start their coursework and placements, the responsibilities and expectations of the job do not match their own expectations. As a result, many drop out of the programs.

Another study [11] indicated that clinical internships and placements might cause confusion and lead to students quitting as nursing tends to follow the apprenticeship learning model: that is, students usually need to follow a group of instructors and leaders for the purposes of knowledge transfer and learning skills. Although textbook knowledge and theoretical understanding are essential, nursing students need to complete year-long internships and placements in hospitals with their supervisors and partners. However, unless secondary school graduates have experienced vocational learning models before, many of them will not understand the apprenticeship learning model. Such confusion may cause misunderstanding, which eventually leads to students dropping out [12].

However, although some students decide to quit nursing programs after commencement, many have no chance to switch to the programs they are interested in due to university regulations and financial limitations. A recent study [13] indicated that nursing students, particularly those in some developing nations, decide to enter nursing training and education programs due to financial and job security considerations. Unlike workers in the private sector, medical and nursing professionals usually have a career development path due to long-term vacancies in the profession. Although some nurses may not enjoy their career development and pathway, due to the social, cultural, and financial background, human resources in the nursing profession are relatively stable [14].

A previous study [15] indicated that Asian students usually select a university major and career path on the basis of external and environmental factors (e.g., financial considerations, recommendations from others, parental influences). In contrast, most westernized students tend to study and enter a profession on the basis of their personal development and interests. For example, a recent research study [16] found that a group of public health students completed their placements in rural communities due to their personal interests and sense of belonging. The study interviewed 58 participants (both students and departmental supervisors). From the negative perspective, many stated that their workload at their placements greatly exceeded their expectations as hospital environments are not the same as the classroom environment. Although all of the students completed the program, it was expected that some might quit the profession due to the mismatch between their experiences, sense of belonging, and career decision-making process.

### 1.2. Purpose of the Study

This study has three aims. First, the medical and nursing professions have always faced significant human resource shortages due to the growing and aging population across the world. In this study, the researcher examined the situation in South Korea. Although South Korea is one of the developed regions in the East Asia region, medical and nursing professionals are still in high demand. Therefore, it is important to establish and develop ways to attract students to join nursing training and education programs in order to fill the gaps.

Second, the experiences, sense of belonging, and career decision-making process of potential student nurses could change due to social, cultural, internal, external, and personal factors. Therefore, it is important to gain an understanding of the experiences and views of nursing students (in this case, in South Korea).

Last but not least, COVID-19 pandemic is one of the significant environmental influences of the past decade. Environmental factors such as the COVID-19 pandemic may influence the experiences, sense of belonging, and career decision-making process of nursing students. Therefore, this study examines the views and opinions of nursing students who have been impacted by the current COVID-19 pandemic and the recent changes in the medical profession and working environment.

In short, this research study was guided by one research question, which was:

How the COVID-19 issue influenced the thinking and decision-making process of these nursing students in South Korea?

## 2. Methods

The employment of the qualitative research method [17,18,19,20] was used for this study. One of the major directions for this study is to gather and capture the lived stories, feedback, understanding, experiences, and sharing [21,22] from a group of pre-service nursing students in South Korea. Therefore, if the researcher employs the quantitative research tools, such as surveys and questionnaires [23] with a large group of people, the researcher may miss some in-depth understanding and sharing [16,24] from the participants. For example, how would the participants describe their feeling and how come the participants decide to conduct some behaviors. Therefore, in order to seek the behavioral sciences and the decision-making process [24,25,26] of the participants, the qualitative research method should be appropriate for this study.

Although the researcher used to plan to collect data information from a single university, feedback and opinions from a certain group of individuals may not reflect the overall performance and situation in the current South Korean environment and society. Therefore, after careful consideration, the researcher decided to expand the population to different nursing schools and universities in order to cover the opinions and voices from a larger population in South Korea [17,19].

The general inductive approach [27] has been employed as a major tool for this study. According to Thomas [27], the general inductive approach is a useful qualitative research method and tool that covered most of the directions and approaches in the fields of education and social sciences [28]. Moreover, without particular limitations and requirements, qualitative researchers could collect data information from the participants with a general perspective [27].

### 2.1. Theoretical Framework: Social Cognitive Career Theory

The Social Cognitive Career Theory (SCCT) was originally created by Lent’s team [29] during the mid-1990s. The theory advocated that individuals’ behaviors are not a result and outcome of individual behaviors and selections. Rather, individuals’ career development and selection are inter-influenced by financial considerations, internal and external environmental factors, educational and career achievements, and personal goals. Based on the theory, individuals’ career development can be impacted by single or multiple factors [30,31].

In order to investigate the study and background, and understand the holistic performance of nursing students’ behaviors and concepts, the Social Cognitive Career Theory (SCCT) has been used for the research [24,25,29,32,33,34,35,36,37,38]. The SCCT has the purpose to explore and investigate the career decision related to academic and career choice chances, the performance, internal, and external factors of individuals [24,25]. The SCCT argued that the background factors of the individuals are social networks, school connections, enrolled schools, peer relationships, financial backgrounds, family situations, living communities, educational backgrounds, place of origin, skin color, gender, and social situations. However, such behaviors can be changed due to internal and external elements, such as time, age, environmental factor, social situation, and even family influence [35,39,40]. Individuals’ decisions and behaviors can be changed and developed due to various situations and personal developments. For example, a previous study [6] indicated that the East Asian tradition about filial piety could be a significant factor for career development and decision among a group of nursing students. Therefore, the SCCT provides the lens and tools for researchers to explore and discover the reasons and motivations of career development and decision, in this case, nursing students.

In this case, the researcher employed this SCCT as the lens to understand and explore how the COVID-19 pandemic influenced the experience, sense of belonging, and career decision-making process of nursing students in the South Korean environment.

### 2.2. Participants

Fifty-eight traditional-age nursing students, regardless of their gender, were invited for this study. All agreed to participate in this study. All the participants were South Korean nations who can speak both Korean and English languages. The participants are currently enrolled at one of the nursing schools within the university system in South Korea. None of them joined any international exchanging programs before the interview sessions. First, the researcher tended to conduct a case study [41] for a small group of people at a single nursing school at a university. Therefore, in order to expand the overall population, the researcher decided to employ the snowball sampling strategy [17,18]. In other words, based on the personal relationship, the researcher interviewed a small group of people. After the first-round interview sessions, each participant was asked to recommend other participants with a similar background (i.e., nursing students). After several rounds of the interview sessions, once the researcher reached the saturation [17,19,42] (i.e., no additional categories and groups are found), the researcher stopped asking additional participants based on the recommendation of snowball sampling strategy. As a result, fifty-eight individuals were invited. The demography of the participants was captured as Appendix A.

### 2.3. Data Collection

Due to the COVID-19 Pandemic, face-to-face interactions, interviews, and even focus group activities were strongly discouraged. Therefore, in order to advocate the ideas of social distancing based on the guideline of the South Korean government, the researcher conducted the data collection sessions via online social media application (i.e., Kakao).

All participants have voluntarily participated in this study. The general inductive approach [27] was employed for meaningful qualitative data collection and analysis. The researcher was the only collector and researcher for the procedure. In order to seek the related information, first, the researcher created an interview protocol with interview questions based on the lens of SCCT [24,25,35,37,43,44,45,46,47]. Unlike other SCCT-based and nurses’ behavioral studies during the normal period, the COVID-19 Pandemic and the related influences may highly impact the experiences, sense of belonging, career decision-making process of individuals. Therefore, the researcher needed to form the particular interview questions based on the impacts of the COVID-19 Pandemic currently. Appendix B indicated the interview questions. It is worth noting that the interview questions were developed based on the SCCT theory [29,32,48] and a recent study [6] about the nursing students’ behaviors.

Second, each participant read the interview questions before the interview session. The participants were invited with an email invitation, content form, and the interview questions. The participants always had at least ten days to read the interview questions before the interview session. Each interview session lasted from 62 min to 89 min. During the interview sessions, the researcher needed to record audio conversations. The participants agreed on the recording.

Third, after the data collection, the researcher transcribed the oral information to written transcripts for reporting. After the written transcripts were created, the researcher sent the related transcript to each participant for the member checking procedure [17,19,25]. All agreed on their sharing and approved the data information.

### 2.4. Data Analysis

After the data collection procedures, more than 600 pages of written transcripts were created based on the semi-structured interviews. Qualitative researchers advocated that large-size data information should be reduced for meaningful themes and subthemes. The researcher followed the recommendation of the general inductive approach [27] for data analysis. For the first-level themes and subthemes, based on the open-coding strategy [17,19], the researcher categorized 12 themes and 24 subthemes for reporting. However, the numbers of these themes and subthemes could not satisfy the intensive nature for reporting. Therefore, based on the recommendation of axial-coding [17,18], the researcher further narrowed the categories into the second-level themes and subthemes. As a result, three themes and five subthemes were categorized for reporting.

### 2.5. Human Subject Protection

All the signed and unsigned content forms, agreements, personal contacts, audio recording, written transcripts, computers, and related materials were locked in a password-protected cabinet. Only the researcher had the rights to read the information. After the study was completed, the researcher destroyed and deleted all the related information immediately due to personal privacy [6,37,49,50,51,52,53].

Due to the content forms and agreements, the name, gender, university name, placement information, and place of origin were masked due to privacy. Due to the intensive networking and population in South Korea, the researcher had the responsibility to protect all the rights of the participants. After careful consideration, the years at university, university location, and the career decision after the COVID-19 Pandemic could be shown.

All subjects gave their informed consent for inclusion before they participated in the study. The study was conducted in accordance with the Declaration of Helsinki, and the protocol was approved by the University Ethics Committee (WSU/2020/01).

## 3. Results and Findings

**Figure 1:**
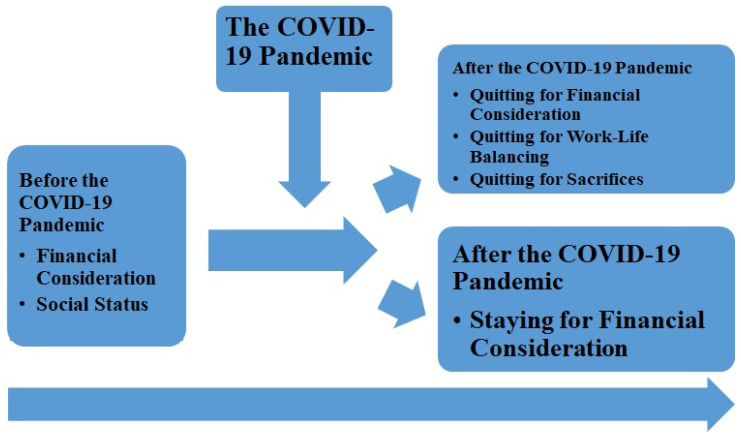
The relationship between the theoretical framework (i.e., the Social Cognitive Career Theory (SCCT)) and the outcomes.

During the interview sessions, the participants answered general semi-structured questions that asked for their opinions, feedback, understanding, and perspectives. Although all of the participants had the same university major and regional background in South Korea, their lived stories, family backgrounds, values, and concepts should not be the same. Figure 1 indicates the relationship between the theoretical framework (i.e., SCCT) and the outcomes of this study.

Unlike previous studies with a wider focus and a more general perspective under normal conditions [13], this study focused on how the COVID-19 issue influenced the thinking and decision-making process of these nursing students in South Korea (i.e., one of the serious regions internationally). In order to help answer the research questions, the findings were categorized into two themes and five subthemes. It was surprising to note that almost all participants were planning to quit their nursing career pathway and profession after completing their university degree due to the COVID-19 pandemic influences. In other words, upon graduation, they would receive a degree in the field of nursing education and registered nurse status but would not be joining the nursing workforce. More importantly, the results showed that all of the students believed that financial considerations and social status always influenced their decision-making process during secondary school and during their current university journey. Table 1 outlines the themes and subthemes of this study.

### 3.1. Before the COVID-19 Pandemic: Financial Consideration as the Major Goal because I Am a Money Person

It was very surprising to find that the COVID-19 pandemic had strongly influenced the experiences, sense of belonging, and career decision-making process of a large number of nursing students in South Korea. The significant results from this study may contribute to an understanding of why such situations arise in the East Asia region, particularly in South Korea.

By listening to the lived stories and life experiences of the participants, the researcher identified several important elements that influenced their decision-making process. The findings of this study revealed that all of the participants intended to complete their university nursing degree but would not be joining the nursing profession after graduation. Using the lens of SCCT [24,25,29,32,33,34,35,36,37,38], the researcher discovered that all the participants had decided to enroll on the nursing education program due to financial considerations. As one participant stated:

I have no personal achievements … no interests … no goals … no career interests … based on my grades. I wanted to study a major that offered a career with a better salary and benefits … [W]orking in a hospital, especially a large-sized hospital in Seoul, is a way of making money … [T]his is the only reason why I wanted to study nursing … [M]oney was the biggest reason …

(P#42)

Another participant stated that she decided to study nursing as her major due to salary considerations:

I could have selected biology or nursing as my major(s) during high school … I selected nursing because nurses can make more money…I wanted to learn biology as it was my interest … but all I need is money and a salary … I would never pick a major that could not produce money …

(P#53, Sophomore)

Although personal interests, goals, and achievements also played a role in their decisions, financial considerations were ranked highest in this study. For example, one participant shared the following:

To be honest, I do not want to be a nurse…but I want to receive a nurse’s salary … I do not really care about the treatment of the patients … I care about myself … I care about money and the holidays … I do not have any dreams … this is Korea … I just care about myself and my money…

(P#54)

#### 3.1.1. Making More Money for Personal Reasons

All 58 participants expressed that financial considerations influenced their experiences, sense of belonging, and career decision-making process as nursing students and pre-service nurses. For example, one participant stated that the significant salary she would receive as a nurse would allow her to buy items, such as handbags, to gain both mental and physical satisfaction:

I can earn a lot of money because I will be working as a nurse in a large hospital … I want to buy a lot of handbags and cosmetic items and a luxury car, and I want to change my cell phone every season … I cannot afford this lifestyle at the moment as I am not rich enough … but once I can earn money as a nurse … I can do something that can satisfy my lifestyle …

(P#22)

Many similar views and stories were captured from the interview sessions. For example, another participant stated that a nurse’s salary would allow her to buy cell phones and computers:

I love up-to-date cell phones and computers…at the moment, I do not want to buy any outdated models … I need to change my electronic items each season…I want to become a nurse … [I]t is totally about money…I do not care about the patients … [A]ll I need is the money … I am not an angel or whatever, I am a money girl.

(P#34)

Based on the lens of SCCT, many of the participants stated that besides allowing them to purchase items that could satisfy their mental and physical needs, a nurse’s salary would also allow them to travel [24,25,29,32,33,34,35,36,37,38]. For example, more than 50 participants stated that they would spend their salary on traveling abroad once they had saved enough:

I can have long holidays and a good salary to pay for my holidays … I plan to have a working holiday after I have worked in a hospital for one or two years … I do not care about how society and communities see me … I have my own life, and my life is about making money with my nursing licence.

(P#12)

#### 3.1.2. Making More Money for Better Family Life

With the reflection of a previous study [3], besides talking about personal satisfaction and enhancement and how a nurse’s salary and benefits would allow them to gain both mental and physical satisfaction, many of the participants stated that they would use a part of their salary for their family [54]. First, many participants expressed that they would use their salary to buy a bigger house for their marriage:

… [M]aking money is my goal … I don’t care about my patients or my career development … [B]eing a nurse is all about money for my marriage … I want to make more money to buy a house after my marriage … [N]ursing is a good way for girls to make money …

(P#3)

Another participant shared similar opinions about how the development of their nursing career would allow them to have a better life after graduation:

… [N]ursing is one of the best occupations to make money…especially for girls in Korea … I want to buy a house for my parents … I will send money to my younger brothers for university … I picked this major because as a girl … I need to find a way to make money … I was thinking about a career in finance … but only a nursing school accepted my application … [I]t is all about money…

(P#25)

Another group of participants expressed that they wanted to build up a small clinic in their rural community as this would be a way to make money and provide health care in their hometown [24]. Based on a previous study [53], although each government district in South Korea has its own public health service, private clinics and hospitals are not uncommon in many regions. The participants believed that they would be able to save enough money to invest in a clinic for profit-making purposes:

... I have a plan with my father already … [A]fter I have gained several years of experience and saved enough money in Seoul … we can invest in a private clinic in the rural community … I already have a plan and timetable … I want to be a boss … I do not want to work for someone … I want to manage the bookkeeping instead of nursing … [M]edical services can be a business …

(P#47)

All 58 participants strongly believed that financial and profit-making considerations were their priorities when choosing a university major and planning their career development [6]. Using the lens of SCCT [24,25,29,32,33,34,35,36,37,38], this researcher found that educational interests and personal interests were less significant for these students; rather, their descriptions of their experiences, sense of belonging, and career decision-making process highlighted the importance of financial considerations.

### 3.2. Before the COVID-19 Pandemic: Climbing the Social Ladder

Based on some previous studies, social status and level are important considerations for many individuals in South Korea in selecting their university major and career development due to traditional Confucian thinking and behavior [55,56,57]. Unlike individuals in westernized communities, who tend to select their university major and career development on the basis of their interests and personal enhancement, individuals in South Korea always regard their university major and career development as tools for social leveling.

A large number of participants stated that being a nurse is one of the best ways for females to escape from poverty as the profession’s selection procedure does not discriminate on the basis of gender. Using the lens of SCCT [24,25,29,32,33,34,35,36,37,38], the researcher captured and gathered some interesting opinions from the participants. However, although this theme mainly focuses on the ideas and themes of social status, many of the ideas were related to financial considerations with elements of social status.

#### 3.2.1. Medical and Nursing as Some of the Highest Occupations in South Korea

*I want to reach the upper level of society with my nursing degree and job … I don’t like nursing and I don’t like caring for patients … but I like the social status … perhaps … because many Korean people believe nurses are upper-level individuals in the society … I want to become one of the highest people in the community … again, I do not like patient caring … I like social level and money …*

(P#33)

In this study, 56 participants stated that nursing is the way to enter the upper level of Korean society. For example, many expressed the view that once they have gained registered nurse status, they can become members of the upper level in society:

… I study nursing only because I want to build up to the upper level … I really hate caring for patients … I will tell my supervisor I am a lazy girl … but society needs me as the there is a shortage of nurses … I can find jobs pretty easily … [A]s a registered nurse, I have an advantage in society … I can gain jobs that I like …

(P#32)

Another participant shared a similar opinion about how registered nursing status could help her to gain nursing jobs in Korea:

… I don’t care about my working environment … I care about how my nursing degree will help me gain a job with less responsibilities … I know nursing is not an easy profession … but I can adjust my workload…I am not here to care for patients … I only want a job so that I can engage in my hobbies and interests after working hours and for the social status I will have as a nurse …

(P#41)

It is clear from their comments that many of the participants believed that they would be able to develop their career in nursing as Korean society is thirsty for registered nurses. Many of the participants selected nursing as their career choice due to financial and social status considerations [24,25,29,32,33,34,35,36,37,38], and most understood how to manage their license and professional registration to attain higher status in society.

In addition to financial considerations and their connection to social status and social leveling, another large group of participants believed that their nursing career might help them to have a better marriage and relationships [58]. For example, one participant indicated that she wanted to use her job as a nurse to marry a rich man:

…I want use my occupation as a nurse as a way to marry a rich organizational leader, as nurses are sexy and attract rich men in the business field … [P]erhaps I can marry a doctor or hospital leader … I don’t want to work as a nurse once I am married … [M]y purpose in choosing to study nursing and enter the profession is to make money … I am not here to care for patients. I am here for the money and social status …

(P#38)

In short, using the lens of SCCT [24,25,29,32,33,34,35,36,37,38], this study revealed that all of the participants indicated the relationship between financial and social status/level considerations and their experiences, sense of belonging, and career decision-making process. The finding from this section is hardly mentioned in the current literature based on a westernized perspective. It is worth noting that all of the participants cared about how their occupation and profession could help them attain some of their financial goals. Most of them stated that they had no strong interest in being a nurse as a lifelong career.

### 3.3. During the COVID-10 Pandemic: Quitting or Staying in the Professional Due to Financial and Work-Life Balance Considerations

#### 3.3.1. Staying in the Profession for Financial and Business Networking Purposes: COVID-19 as a Provider of Business Opportunities

Although individuals’ decisions can change due to unforeseen circumstances [59], many individuals have firm views with regard to their university major and career development [60]. For example, one participant stated that she wants to become a nurse due to financial considerations:

I need to make more money, so I need to become a nurse. Although I really don’t like patient caring … I need to make more money for my dream traveling … and perhaps handbags and some leisure activities … I just hate patient caring and I hate taking care of people …

(P#25)

Moreover, some participants stated that their occupation as a nurse would allow them to attain higher-level status in South Korean society:

Korean people like nurses … because they are working in higher status workplaces … such as hospitals … I like this prestige … [I]t is my first priority … in seeking to become a nurse … [I]t is not about the patients …

(P#49)

However, in this study, under the influences and impacts of the COVID-19 pandemic, only two participants expressed a firm interest in becoming a nurse after graduating from university. Based on the lens of SCCT [24,25,29,32,33,34,35,36,37,38], the researcher captured many messages about how the COVID-19 pandemic had influenced the participants’ experiences, sense of belonging, and career decision-making process. In particular, from the two participants above, the researcher gleaned some significant reasons for their decision to continue the development of their nursing career. In short, these two participants strongly believed that financial considerations outweighed any issues associated with COVID-19 and its related consequences:

I decided to study nursing because I want to make more money and I want to save more money for traveling and shopping … for handbags and leisure activities … The COVID-19 pandemic will not change my mind about this money-making decision … I still want to make money from the medical field … I will start my own clinic … It should be a way to make money …

(P#50)

Another participant shared a similar opinion about financial considerations and social status and leveling, stating that the COVID-19 pandemic offers an opportunity to make money due to the increased awareness of and need for personal care and personal hygiene:

… I am sure I will not leave the medical profession … I will use this opportunity to make more money from patients … I have a business mind with a nursing focus … I will enter a hospital … to understand how it operates and patients’ behaviors … [A]fter gaining several years’ experience … I will start my own clinic…but again, I need to make money … [T]he COVID-19 pandemic will not change my focus on money …

(P#11)

Their stories reflect the finding of several previous studies that once individuals understand their career perspective, only a few internal and external elements and factors can influence or change their behaviors [24,25,29,32,33,34,35,36,37,38].

#### 3.3.2. The Insignificant Salary and Benefits in the Nursing Profession Do Not Match the Sacrifice

First, due to the influences of the COVID-19 pandemic, many participants stated that the high-risk career pathways in the medical and nursing profession always outweighed their financial needs, demands, and objectives. Using the lens of SCCT [24,25,29,32,33,34,35,36,37,38], the researcher gathered some opinions from the participants. For example, how COVID-19 and financial considerations can be combined in the career decision-making process. One group of participants stated that they intended to quit the nursing profession because of the long working hours of medical and nursing professionals and the lack of overtime salary payments and compensation:

… I joined the nursing profession because of the salary. I do not have money to start my clinic in the future…I have to work in a hospital to finance my leisure activities and handbags … Although the salary is very attractive, I have to work for more than 10 h a day … but I do not receive any additional payments even when I work overtime…I disagree with this situation …

(P#16)

With the reflection of some previous studies, besides the lack of overtime payments and the workload due to the COVID-19 pandemic and social situation internationally, many participants stated that the long working hours had an impact on their work-life balance [61,62]. For example, one group of participants commented that they cannot go shopping when they have to do overtime:

… [M]y hobby is not nursing, caring for patients, or working for society … [M]y hobbies are buying handbags…watching movies … going to a coffee shop and chatting with my friends … [D]ue to this COVID-19 pandemic, many medical professionals need to work overtime with no additional salary … [If] I cannot go to buy a handbag after I finish work, I will not join this profession …

(P#39)

Many participants indicated that the mandatory and voluntary-based overtime responsibilities in the medical and nursing professions did nothing to increase their interest in working in these professions due to the insignificant salary [16,63]. Although some participants understood this situation before the interview sessions, the COVID-19 pandemic had led many to reconsider their experiences, sense of belonging, and career decision-making process through the lens of SCCT. For example, many believed that they and their family members should receive free plastic surgery at least once per year in their hospital. However, due to the COVID-19 pandemic, many medical organizations had rearranged their insurance plans and benefit packages due to financial stresses. As a result, many of the participants had lost interest in joining the profession:

My goal is to become a sexy nurse K-pop star who can make a lot of money on the stage, TV shows, and movies … [D]on’t you think the gimmick of sexy nurse and K-pop is more attractive than a fat nurse working in a hospital for 30 years? … I want to work in a plastic surgery clinic in the future because I want to take advantage of the free plastic surgery … but for now, I will just follow the K-pop direction after my graduation…

(P#51)

The idea of taking advantages and benefits from employers was not confined to one participant. Many expressed a similar view about their intentions. Another participant also shared an idea about how COVID-19 influenced the benefit packages, which impacted their experiences, sense of belonging, and career decision-making process:

I was planning to work just in surgery before I left the hospital … but I don’t think that will work after the COVID-19 pandemic … [M]any hospitals and medical organizations have changed their benefit packages … no more plastic surgery benefits for staff with less than five years’ working experience … I may enter the business industry, for example, promotion or TV show work, with my professional nursing skills.

(P#2)

Many indicated that if they cannot be given additional benefits and advantages in hospitals and organizations, they would be interested in joining other professions [64]. Currently, due to the COVID-19 pandemic, many organizations have rearranged their benefits packages due to financial stresses. The motivations for joining the nursing profession have thus been reduced as such changes have not had any positive impacts on the benefits offered.

More than 45 participants stated that they did not want to work as assistants to others but as leaders and employers in business teams and companies. More importantly, many indicated that they disliked helping patients with special needs. For example, one participant stated that, in their opinion, helping patients is not a positive work responsibility:

I am planning to work as a leader or join a leadership team … I do not want to help any patients as I want to lead people and make more money with my skills … Since the COVID-19 pandemic, I have changed my mind somehow … I will not join the nursing profession … [I]nstead, I want to use my nursing and medical skills to start a YouTube channel for health promotion …

(P#44)

More than half of the participants stated that they did not want to touch patients’ bodies as many patients may be infected by the COVID-19 virus. One participant made this significant comment:

I am sure that I am not going to touch those people who are infected with this international virus … I disagree with how the government treats nursing professionals … [W]e are human and we have family members … [W]hy would the government send us to the frontline for the patients? I want to make money and manage people …

(P#33)

Another participant stated:

… [I]n the first place, I do not have a strong interest in nursing, but I am interested in money, my name, a good position, and a good company … [C]aring for patients and older people is of no interest to me … [A]fter this COVID-19 pandemic, I do not think I will join the nursing profession as I really don’t want to provide care to older people …

(P#21)

In conclusion, through the lens of SCCT [24,25,29,32,33,34,35,36,37,38], the sharing of the participants revealed how financial considerations influenced their experiences, sense of belonging, and career decision-making process. Although the participants planned to change their career direction from nursing to other professions, such as business and the entertainment industry, the original SCCT factor (i.e., financial considerations) did not change significantly. Financial considerations and money-making ideas always influenced their behaviors and decisions. However, due to the COVID-19 pandemic, their experiences, sense of belonging, and career decision-making process had changed slightly due to the insignificant salary and unattractive benefits in the nursing profession and the nature of the profession. Similar findings have also been reported in several previous studies. However, individuals are less likely to change their original goals and personal intentions in regard to the direction of their career development (i.e., money-making ideas).

## 4. Discussion

The original purpose of this study was to capture the experiences, sense of belonging, and career decision-making process of university nursing students in South Korea. As the COVID-19 pandemic has influenced the medical and social care professions across the world, the researcher sought to discover how the pandemic has influenced the overall performance and perspective of pre-service medical professionals in the field. Using the lens of SCCT [24,25,29,32,33,34,35,36,37,38], the researcher discovered that all the participants had decided to enroll on the nursing education program due to financial considerations. The results of this study support the ideas and concepts from SCCT [24,25,29,32,33,34,35,36,37,38] that suggest that individuals’ experiences, sense of belonging, and career decision-making process can be influenced by financial considerations. In this study, financial considerations were found to have a significant impact on most of the participants.

Based on the lens of SCCT, they all stated that they decided to become registered nurses due to financial considerations [24,25,29,32,33,34,35,36,37,38]. Although some shared some insignificant comments about personal goals and family influences, all of them spent most of the time talking about financial considerations, money matters, and social status. At the personal level [24,25,29,32,33,34,35,36,37,38], different individuals expressed various ideas about how being in a nursing position would allow them to buy and acquire items and a sense of personal belonging in order to achieve mental and physical satisfaction. In short, based on the lens of SCCT [24,25,29,32,33,34,35,36,37,38], this theme indicated the financial considerations and reasons why these participants intended to become nurses after university graduation. Using the lens of SCCT [24,25,29,32,33,34,35,36,37,38], this researcher found that educational interests and personal interests were less significant for these students; rather, their descriptions of their experiences, sense of belonging, and career decision-making process highlighted the importance of financial considerations.

Using the lens of SCCT [24,25,29,32,33,34,35,36,37,38], in this study, all 58 participants expressed different opinions about how the medical profession and a nursing job would help them to achieve higher social status as women in Korean society. Based on a previous study [56], in the current social environment in South Korea, discrimination due to place of origin is not uncommon, particularly for individuals and females who come from poor or rural communities. From traditional East Asian perspectives, the medical and nursing professions are among the highest occupations in society due to their nature [65]. Although youths in South Korea do not have many life and working experiences, many understand and experience social expectations and social leveling as university students. In fact, given traditional East Asian perspectives, members of the general public view nurses as caregivers who can play a positive role in caring for the family, elderly, and children. Based on the lens of SCCT, all of the participants stated that their occupation could help them in dating and having relationships with rich men [24,25,29,32,33,34,35,36,37,38].

Individuals’ experiences, sense of belonging, and career decision-making process are usually solidly based on the elements described in SCCT [24,25,29,32,33,34,35,36,37,38]. Many previous studies [24,25,29,32,33,34,35,36,37,38] have indicated that although the environment elements, socio-cultural background, family situation, and financial elements are changed within the individuals’ background and environment, many individuals continued to work within their related industry as they have already established their sense of belonging and career decision-making process. Although individuals’ decisions can change due to unforeseen circumstances [59], many individuals have firm views with regard to their university major and career development [60].

However, in this study, under the influences and impacts of the COVID-19 pandemic, only two participants expressed a firm interest in becoming a nurse after graduating from university. Based on the lens of SCCT [24,25,29,32,33,34,35,36,37,38], the researcher captured many messages about how the COVID-19 pandemic had influenced the participants’ experiences, sense of belonging, and career decision-making process. In short, these two participants firmly believed that the COVID-19 pandemic will offer them an opportunity to start their own business on the basis of their nursing degree, registered nurse status, and experience in a hospital environment after their graduation. Viewed through the lens of SCCT [24,25,29,32,33,34,35,36,37,38], these two participants understood how financial considerations influenced their experiences, sense of belonging, and career decision-making process.

Although a few participants stated that staying in the medical and nursing professions would offer them business opportunities due to the COVID-19 pandemic, almost all of the nursing students had decided to quit the nursing profession after graduating from university. Several factors emerged from their responses, the main ones being the insignificant salary, unattractive benefits, and nature of the nursing profession [55]. Almost all of the participants expressed the view that the benefits of working in the medical and nursing professions are not as attractive as those offered by other large organizations or by self-employment [66]. Based on the lens of SCCT [24,25,29,32,33,34,35,36,37,38], the final significant opinion to emerge concerned the nature of the nursing profession. Although the roles and status of nursing professionals have increased, nursing professionals are considered as assistants to doctors and therapists in medical environments [67].

### Limitations and Future Developments

Every study has its own limitations. First, due to the COVID-19 Pandemic and the recommendations of social distancing, no face-to-face interview sessions and focus group activities were conducted [68,69,70]. Although social media technology connected the researcher and the participants, the in-depth sharing and understanding [22,71,72,73] were not able to capture. However, this study can become a blueprint for researchers and scholars to polish the interview skills and interactions between researchers and participants via technology [74,75].

Second, this study only focused on the problems in the East Asian region, particularly in South Korea [55]. However, as the COVID-19 Pandemic is an international medical disaster, other countries must face a similar problem. Therefore, future research can seek the experiences, sense of belonging, and career decision-making process of nursing students or other personnel in any fields [2,76,77]. In fact, the ideas about experiences, sense of belonging, and career decision-making process can be wide due to the influence of the COVID-19 Pandemic. Researchers should not limit the research directions in the field of nursing and the medical profession [2,76,77].

## 5. Conclusions

To the best of the knowledge, this is one of the very first nursing research studies about the experiences, sense of belonging, and career decision-making process of contemporary nursing students [6] under the impacts of the COVID-19 Pandemic in the East Asian region, particularly in the South Korean environment. In fact, during the data collection and analysis period, the COVID-19 Pandemic is still an international medical disaster. This study captured the sharing of the experiences, sense of belonging, and career decision-making process from the nursing students [6,68,69,70], which may high reflect the overall performance and understanding of nursing and medical students with a similar background.

With the lens of the SCCT [24,25,35,37,43,44,45,46,47], the researcher concluded that the participants were mainly influenced by financial consideration [24,35,37]. Although almost all do not want to pursue a nursing occupation after university graduation, this extreme situation allowed the university administrators, hospital leaders, policymakers, human resources professionals, and researchers to plan their future nursing workforces immediately as a group of nursing students will not join the profession after the university graduation due to the influences of the COVID-19 Pandemic. Nevertheless, it is also important for all interested parties to understand how the COVID-19 may change the international orders and career decisions of traditional-age students [34,46]. Although the research study only focused on the ideas of nursing students, related professionals may take this result as the opportunity and blueprint to reform their current planning.

### Practice Implications

There are two recommendations for the practices. First, the current COVID-19 pandemic may increase the turnover rate and stress of both pre-service and in-service public health professionals in both hospital and clinics environment. Therefore, based on the lens of the SCCT and the perspectives of the participants of this study, policymakers, school leaders, hospital leaders, human resource planners, public health professionals, and researchers should take this study as the opportunity to reform and polish their current human resources planning.

Second, based on the results of the current study with the lens of SCCT, many pre-service nursing professionals may not join the nursing profession after university graduation. Therefore, policymakers, government leaders, hospital leaders, and human resources planners should start to recruit and train additional nursing and public health professionals in order to fill up the gaps for the future missing workforce.

## Figures and Tables

**Table 1:** Themes and subthemes.

Themes and Subthemes		
Section 3.1		Before the COVID-19 Pandemic: Financial Consideration as the Major Goal because I am a Money Person
	Section 3.1.1	Making more Money for Personal Reasons
	Section 3.1.2	Making more Money for Better Family Life
Section 3.2		Before the COVID-19 Pandemic: Climbing the Social Ladder
	Section 3.2.1	Medical and Nursing as Some of the Highest Occupations in South Korea
Section 3.3		During the COVID-19 Pandemic: Quitting or Staying in the Profession due to Financial and Work-Life Balance Considerations
	Section 3.3.1	Staying in the Profession for Financial and Business Networking Purposes: COVID-19 as a Provider of Business Opportunities
	Section 3.3.2	The Insignificant Salary and Benefits in the Nursing Profession do not Match the Sacrifice

## Appendix A

**Table A1:** Demography of the Participants.

Name	Years at University	University Location	Career Decision after the COVID-19 Pandemic
P#1	Freshmen (1st Year)	Seoul	Will not enter the nursing profession after university graduation
P#2			
P#3			
P#4			
P#5			
P#6		Busan	
P#7			
P#8		Daejeon	
P#9			
P#10			
P#11	Sophomore (2nd Year)	Seoul	
P#12			
P#13			
P#14			
P#15			
P#16			
P#17		Gangwon-do	
P#18			
P#19		Gyeonggi-do	
P#20			
P#21		Daejeon	
P#22			
P#23			
P#24			
P#25			
P#26	Junior (3rd Year)	Seoul	
P#27			
P#28			
P#29		Jeolla-do	
P#30			
P#31			
P#32		Gyeongsang-do	
P#33			
P#34			
P#35		Daegu	
P#36			
P#37			
P#38			
P#39	Senior (4th Year)	Seoul	
P#40			
P#41			
P#42			
P#43			
P#44		Gwangju	
P#45			
P#46			
P#47		Incheon	
P#48			
P#49			
P#50		Daejeon	
P#51			
P#52		Ulsan	
P#53			
P#54		Busan	
P#55			
P#56			
P#57			Stay in the nursing professional after university graduation
P#58			

## Appendix B

Interview Protocol/Interview Questions Based on the lens of Social Cognitive Career Theory.

Questions in general before the influences of the COVID-19 Pandemic:

(1) Was nursing your first choice for university major? Can you tell me more about your selection procedure?
(2) Why do you want to become a nursing student? What are your motivations? Can you tell me more with examples?
(3) Do you want to become a nurse or medical professional in the medical field? Why or why not?
(4) If you have a chance again, do you want to study nursing as your university major? Why or why not?

Questions about how the COVID-19 Pandemic influenced the experiences, sense of belonging, and career decision-making process:

(5) Did you change your mind about being a nursing student during the COVID-19 Pandemic? How?
(6) How would you describe your experiences, sense of belonging, and career decision-making process due to the COVID-19 Pandemic?
(7) Do you still want to become a nurse after the COVID-19 Pandemic? Why or why not?
(8) What motivations or reasons have been changed after the COVID-19 Pandemic? In any directions, can you tell me more?
